# Design, preparation, and selection of DNA-encoded dynamic libraries†
†Electronic supplementary information (ESI) available: Materials and general methods, experimental details, library selection and sequencing methods and fold enrichment calculations. See DOI: 10.1039/c5sc02467f



**DOI:** 10.1039/c5sc02467f

**Published:** 2015-09-11

**Authors:** Gang Li, Wenlu Zheng, Zitian Chen, Yu Zhou, Yu Liu, Junrui Yang, Yanyi Huang, Xiaoyu Li

**Affiliations:** a Key Laboratory of Bioorganic Chemistry and Molecular Engineering of the Ministry of Education , Beijing National Laboratory of Molecular Sciences (BNLMS) , College of Chemistry and Molecular Engineering , Peking University , Beijing , China 100871 . Email: xiaoyuli@pku.edu.cn; b Key Laboratory of Chemical Genomics , School of Chemical Biology and Biotechnology , Peking University Shenzhen Graduate School , Shenzhen , China 518055; c Biodynamic Optical Imaging Centre (BIOPIC) and College of Engineering , Peking University , Beijing , China 100871

## Abstract

We report a method for the preparation and selection of DNA-encoded dynamic libraries (DEDLs). The library is composed of two sets of DNA-linked small molecules that are under dynamic exchange through DNA hybridization. Addition of the protein target shifted the equilibrium, favouring the assembly of high affinity bivalent binders. Notably, we introduced a novel locking mechanism to stop the dynamic exchange and “freeze” the equilibrium, thereby enabling downstream hit isolation and decoding by PCR amplification and DNA sequencing. Our DEDL approach has circumvented the limitation of library size and realized the analysis and selection of large dynamic libraries. In addition, this method also eliminates the requirement for modified and immobilized target proteins.

## Abstract 2

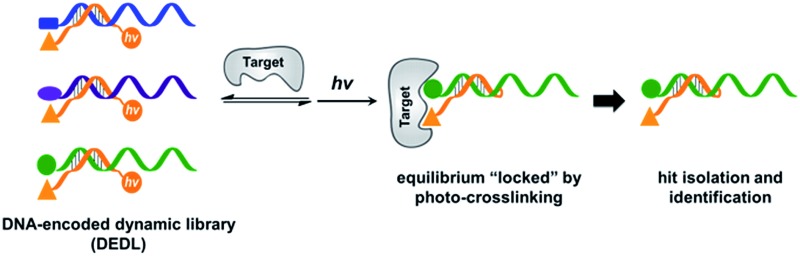
DNA-encoded dynamic libraries (DEDLs) are realized by dynamic DNA hybridization and a novel equilibrium-locking mechanism.

## Introduction

Dynamic combinatorial chemistry (DCC) employs reversible bond formation to create dynamic systems of continuous inter-exchanging chemical entities.^1–4^ Built on the principle of DCC, dynamic combinatorial libraries (DCLs) have emerged as efficient tools for discovering novel ligands for biological targets.^5–8^ Compared with a static library, a DCL has two advantages. First, a DCL allows for a spontaneous library synthesis based on the inter-conversion of compounds through reversible reactions among building blocks (BBs); the entire library can be synthesized by simply mixing the BBs without the need for spatial separation. Second, a DCL is adaptive: adding the target induces the selection pressure to redistribute the BBs, favouring the synthesis of target-binding compounds at the expense of non-binding ones.^9–12^ Moreover, after reaching a new equilibrium in the presence of the target, the library can be “frozen” by stopping the dynamic exchange (*e.g.* by adding an additive or changing the pH to stop reversible reactions), so that the library population change is preserved and ready for subsequent hit identification.^1,6^ DCLs have shown great potential in accelerating the discovery of lead compounds in drug discovery,^5,6,13,14^ such as in fragment-based^15–18^ and structure-based drug design.^5,19,20^

However, DCLs face a major limitation of low library diversity, mainly resulting from the lack of suitable analytical methods. Typically, chromatographic methods, such as HPLC, are used to resolve DCLs and to identify binders by comparing spectra with and without the target,^18,21–23^ but HPLC does not have the capacity to resolve large libraries containing many different compounds.^16,24^ Other methods, such as non-denaturing mass spectrometry,^25^ NMR,^26^ and spectroscopic methods (UV and fluorescence)^27–29^ have been employed for DCLs, but the resolution and throughput of these methods are also not sufficient for large libraries. Otto, Miller, and their respective co-workers have developed several elegant approaches capable of analyzing and selecting large DCLs (∼10 K compounds);^29–32^ however, in most cases, DCLs only contain 10–100 compounds. Since the probability of discovering high affinity ligands increases with the library diversity, the limitation of the library size has presented a significant obstacle for DCLs.^23^ New approaches capable of resolving and analyzing large DCLs are still highly desired.

**Fig. 1:**
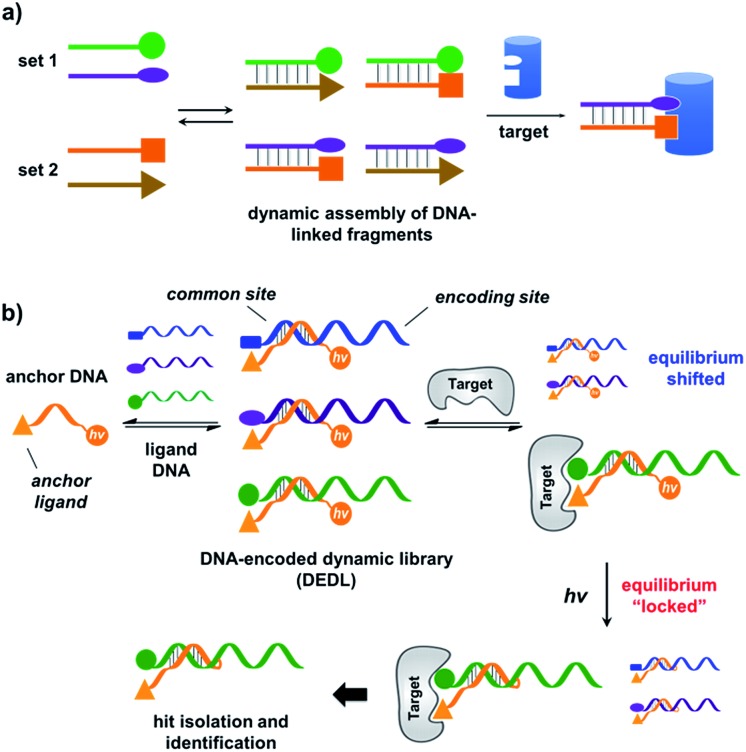
(a) Previous work: two sets of DNA-linked fragments form dynamically exchanging duplexes; addition of the target enriches high affinity duplexes.^49,67,68^ (b) DNA-encoded dynamic library (DEDL) (this work): an anchor DNA forms dynamic duplexes with multiple ligand DNA strands, forming the library. Adding the target shifts the equilibrium, favouring the formation of high affinity binders. A photo-crosslinker in the anchor DNA locks the shifted equilibrium under irradiation. Crosslinked binders can then be isolated for hit identification *via* PCR amplification and DNA sequencing.

A DNA-encoded library (DEL), in which each compound is linked with a unique DNA tag, is another combinatorial library approach employing mixed compounds in library processing.^33–42^ In contrast to DCLs, due to DNA's high encoding capacity, DELs can contain millions of different compounds;^43–46^ library selection can be feasibly decoded using PCR amplification and DNA sequencing.^47,48^ Therefore, introducing DNA-encoding to DCLs could be an effective strategy to address the limitation of their library size. Previously, nucleic acids have been successfully used as programmable templates or scaffolds with spatial precision to display ligand combinations interacting with various biological targets.^49–66^ The Neri group developed a method named an Encoded Self-Assembling Combinatorial (ESAC) library, in which two sets of DNA-linked fragments form a static library by combinatorial duplex formation.^65,66^ Hamilton and co-workers introduced dynamic exchange in DNA hybridization, so that the target can shift the equilibrium and enrich high affinity fragment combinations (Fig. 1a).^49,67^ Very recently, Zhang and co-workers reported a similar system achieving target-induced enrichment of DNA duplexes.^68^ These studies have nicely shown that the principle of dynamic exchange can be applied to DELs; however, more systematic methodology for the preparation and selection of DNA-encoded dynamic libraries (DEDLs) has yet to be developed. Moreover, previous studies require modified and immobilized targets in library selection, which is not compatible with proteins that are difficult to purify or modify, such as membrane proteins.^69,70^ Aiming to address these issues, here we report the detailed study of a DEDL system, including library preparation, encoding, selection, hit deconvolution, and notably, a novel “locking” strategy to freeze the equilibrium shift for hit isolation and identification.

## Results and discussion

Our strategy is shown in Fig. 1b. Libraries of BBs are conjugated to different DNA strands (ligand DNA), all having a common sequence that can form dynamically exchanging duplexes with an “anchor DNA”, which is conjugated with an “anchor” molecule. Upon target addition, the equilibrium shifts to form more high affinity bivalent duplexes. Next, the photo-reactive group on the anchor DNA can crosslink the two DNA strands upon irradiation, thereby stopping the dynamic exchange and locking the shifted equilibrium. The distal region on the ligand DNA encodes the BB's chemical identity, and the crosslinked duplex can be isolated for hit identification with PCR amplification and DNA sequencing (Fig. 1b). By combining the features of DELs and DCLs, our design allows for the selection of high diversity DCLs to discover synergistic fragments for “affinity maturation” of the anchor molecule.^65,66,71,72^

**Fig. 2:**
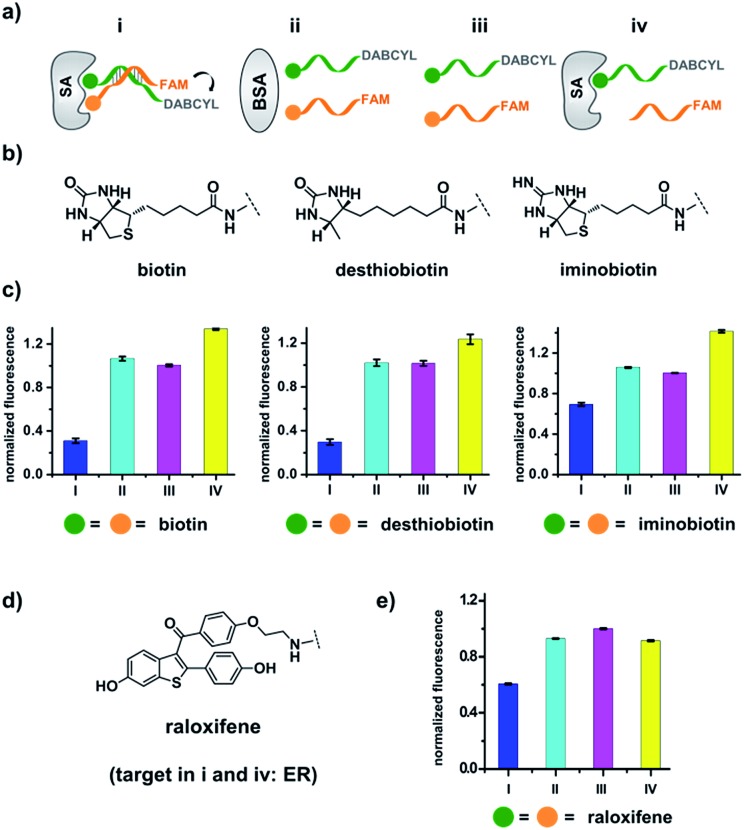
(a) Two complementary DNA strands conjugated to biotin, desthiobiotin, or iminobiotin and FAM/DABCYL groups were mixed with the target SA (**i**), with BSA (**ii**), with no protein (**iii**), or with one ligand omitted (**iv**). (b) Structures of the small molecule ligands. (c) Fluorescence quenching results. FAM fluorescence values were measured and normalized to (**iii**). Left panel: with biotin; middle panel: with desthiobiotin; right panel: with iminobiotin. (d) Structure of the raloxifene ligand. (e) Fluorescence quenching results of the raloxifene–ER system. ER was used as the target in (**i**) and (**iv**). DNA: 200 nM each; protein: 400 nM. The DNA and protein were incubated at 30 °C for 1 h before measurement using a fluorophotometer. Excitation: 494 nm; emission: 522 nm. Error bars (standard deviation, SD) are based on three replicates of each experiment.

We first verified that dynamic DNA duplex formation can be affected by the target protein.^49,68^ As shown in Fig. 2a, a fluorescein (FAM) molecule and a quencher (DABCYL) were conjugated to two complementary DNA strands; the decrease of fluorescence therefore indicates DNA hybridization. The other end of the DNA was conjugated to a biotin, a desthiobiotin, or an iminobiotin molecule (Fig. 2b). These ligands are well known to bind to adjacent pockets on the tetrameric protein streptavidin (SA) with different affinities (*K*_d_: 40 fM, 2.0 nM and 50 nM, respectively).^73^ Moreover, we reason that, in order to establish dynamic exchange, the DNA duplex should have a melting temperature (*T*_m_) close to the experiment temperature, and it should also be sufficiently long to ensure hybridization specificity; therefore, either 6- or 7-base DNA duplexes were chosen in our study.

**Fig. 3:**
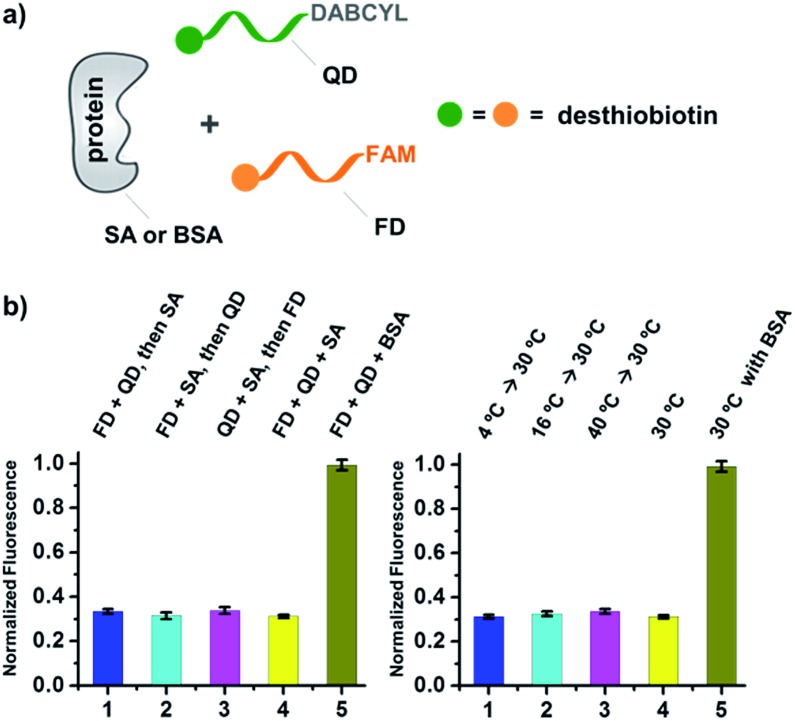
(a) Two desthiobiotin-labelled DNA strands conjugated to FAM or DABCYL were mixed with SA or BSA in different orders or at different temperatures; fluorescence decreases were then measured. (b) Left panel: data from different mixing orders. Right panel: data from different temperatures; SA was used as the target except in 5 where BSA was used as a negative control. The experimental conditions were the same as those for Fig. 2 except for the mixing order and temperature. Error bars (standard deviation, SD) are based on three replicates of each experiment.

As shown in Fig. 2c, for all three ligands the fluorescence decreased significantly in the presence of SA, suggesting the formation of the ternary complex (**i**). In contrast, in control experiments with the non-specific protein BSA (bovine serum albumin) (**ii**), without SA (**iii**), or with one ligand omitted (**iv**), little or no fluorescence decrease was observed, indicating the quenching in (**i**) depends on specific bivalent binding to SA. Notably, ∼40% quenching was observed for the weak binder iminobiotin (Fig. 2c, right panel). Furthermore, we performed similar fluorescence quenching experiments with raloxifene, an estrogen receptor (ER) modulator (Fig. 2d);^74^ dimeric raloxifene ligands are able to bind to the two binding pockets on estrogen receptor dimers.^61,75,76^ Similar to the biotin ligand series, a significant fluorescence decrease was observed in the presence of the specific target ER and the bivalent raloxifene duplex (Fig. 2e). In addition, as a thermodynamically-controlled system, an important feature of DCLs is that the same state of equilibrium can be reached from different starting points.^77,78^ In order to verify this, we either altered the mixing order or incubated the mixture at 4 °C, 16 °C, 30 °C or 40 °C for 30 min before incubation at 30 °C for another hour (**QD** and **FD**; Fig. 3a). We observed that all experiments reached the same equilibrium based on fluorescence readings, proving the dynamic nature of our system (Fig. 3b).

**Fig. 4:**
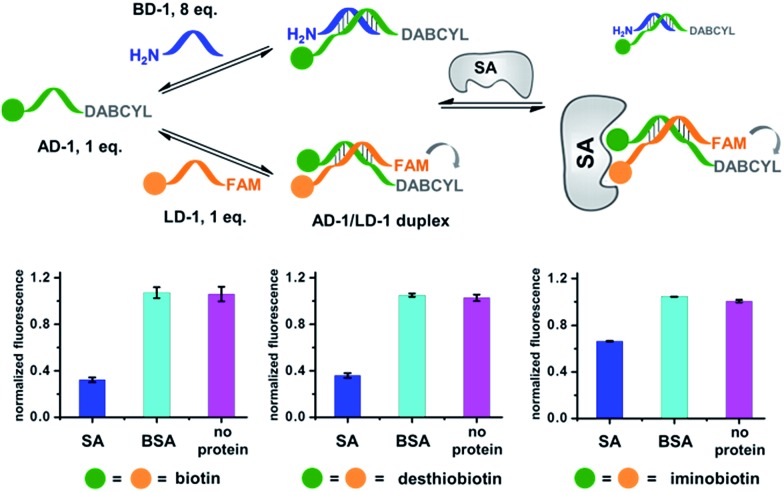
Verification of the target-induced equilibrium shift, determined by a fluorescence decrease. Fluorescence values were normalized to the “no protein” experiment. **AD-1** and **LD-1**: 200 nM; **BD-1**: 1.6 μM; proteins: 400 nM. The experimental procedures were the same as those for Fig. 2. Error bars (standard deviation, SD) are based on three replicates of each experiment.

Next, we investigated whether the target has shifted the equilibrium to promote the assembly of high affinity duplexes. As shown in Fig. 4, we mixed a non-fluorescent background DNA (5′-GTCTGC-3′-NH_2_; **BD-1**) with a fluorescent ligand DNA (5′-FAM-GTCTGC-3′-ligand; **LD-1**) at an 8 : 1 ratio. Both DNA strands dynamically compete for hybridization to **AD-1**, which is conjugated to an anchor ligand and a DABCYL quencher (5′-ligand-GCAGACT-3′-DABCYL). The bivalent **LD-1**/**AD-1** duplex is expected to have a higher affinity for SA than the monovalent **BD-1**/**AD-1** duplex. After mixing the DNA strands (**BD-1**/**LD-1**/**AD-1**: 8 : 1 : 1) with SA, we observed significant fluorescence quenching for all three ligands, indicating the equilibrium has been shifted to favour the formation of the (**LD-1**/**AD-1**)-SA ternary complex.

As a negative control, the non-binding BSA did not shift the equilibrium (cyan columns; Fig. 4). These results have demonstrated that the target can indeed promote the assembly of high affinity binders.

**Fig. 5:**
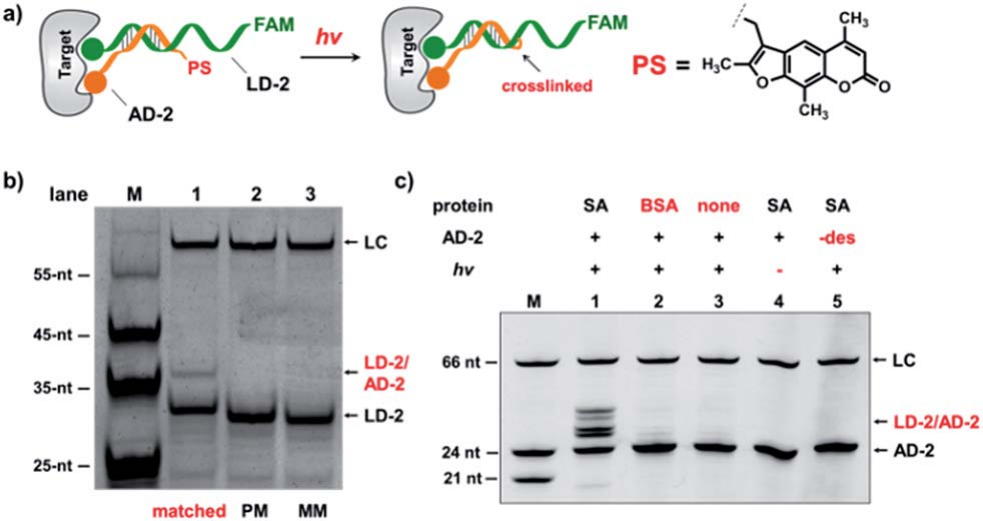
(a) Psoralen (PS) was chosen to lock the equilibrium in the DEDL. (b) Three sets of **AD-2**/**LD-2** were mixed, irradiated, and analysed by denaturing electrophoresis. PM: partially mismatched (2-base mismatch); MM: fully mismatched; LC: a 66-nt DNA loading control. (c) 5′-Desthiobiotin-labeled **LD-2** and **AD-2** were mixed, irradiated under different conditions, and then analysed by denaturing electrophoresis (18% TBE–urea denaturing PAGE). Lane 1: with SA; lane 2: with BSA; lane 3: no protein added; lane 4: no irradiation; lane 5: no desthiobiotin on **AD-2**. **AD-2**: 300 nM; **LD-2**: 200 nM; irradiation: 365 nm for 30 s at 30 °C using a UV LED point light system; short irradiation reduces non-specific crosslinking in the background. M: marker; –des: no desthiobiotin.

In the selection of DCLs, it is often necessary to stop the dynamic exchange and “freeze” the shifted equilibrium, so that the library population change, induced by the target, can be preserved for further characterization. For example, adding NaBH_3_CN to reduce imines to stable amines is a popular method to stop the dynamic imine formation,^21,22,79–81^ and lowering the pH can effectively disable disulfide exchange and reversible Michael addition, which optimally occur at basic pH.^16,17,24,31,78^ In this study, we designed a novel photo-crosslinking strategy to stop the dynamic DNA duplex exchange. Photo-crosslinking is kinetically fast and can be imposed/withdrawn conveniently with minimal perturbation to the system.^82^ As shown in Fig. 5a, psoralen (PS), a photo-crosslinker widely used in nucleic acid crosslinking,^83–86^ was conjugated to the 5′-end of a short 7-nt DNA bearing the anchor molecule (**AD-2**). **AD-2** is complementary to the 5′-end of a 24-nt DNA having a ligand and a FAM group (**LD-2**). Moreover, **LD-2** also contains a thymine group at the site opposite to PS, which is known to be able to improve the crosslinking efficiency.^87^ After DNA incubation and target addition, irradiation triggers crosslinking between **AD-2** and **LD-2**, thereby stopping strand exchange and locking the equilibrium. The crosslinked **AD-2**/**LD-2** duplex can then be isolated for PCR amplification and DNA sequencing to decode the ligand synergistically binding to the target with the anchor molecule. First, we prepared fully matched, partially mismatched, and fully mismatched **AD-2**/**LD-2** duplexes. These DNA duplexes were mixed, irradiated, and analysed by denaturing electrophoresis. The crosslinked product was only observed with the fully matched DNA duplexes (lane 1; Fig. 5b). Next, a set of desthiobiotin-labelled **AD-2** and **LD-2** strands was subjected to the same procedure; results show that only in the presence of SA was the crosslinking product detected (lane 1; Fig. 5c). Multiple bands appeared in lane 1 of Fig. 5c; mass analysis confirmed that all are crosslinked duplexes (see the ESI†). We hypothesize that the “T” shape of the crosslinked duplex may partially renature in the gel, a phenomenon that we have observed previously.^88^ In all negative controls (with BSA, no protein, no irradiation and no desthiobiotin on **AD-2**; lanes 2–5, Fig. 5c), no or very little crosslinking was detected. The product bands were excised, extracted, and quantified. With SA, a 40% crosslinking yield was obtained. Collectively, these results have demonstrated the specificities of PS-based interstrand DNA crosslinking and its suitability for capturing target-induced duplex formation.

**Fig. 6:**
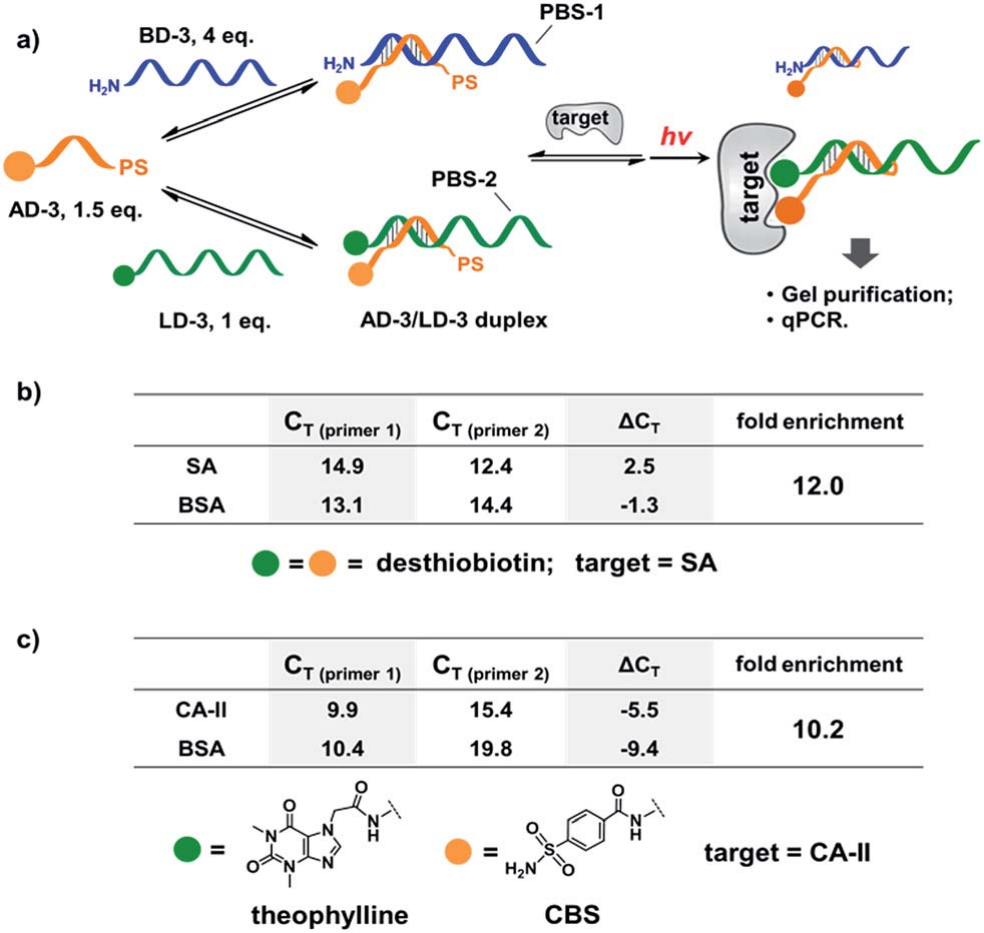
(a) PS-based photo-crosslinking locks the shifted equilibrium for the subsequent hit isolation and qPCR analysis. After gel-purification of the crosslinked duplexes, qPCR was performed to determine the *C*_T_ values and to calculate the fold enrichment of the **AD-3**/**LD-3** duplex. (b) Results of the bivalent desthiobiotin–SA system. (c) Results of the theophylline/CBS–CA-II system. Δ*C*_T_ = *C*_T (primer 1)_ – *C*_T (primer 2)_. **AD-3**: 300 nM; **LD-3**: 200 nM; primers: 200 nM. The experimental procedure was the same as that for Fig. 5. See the ESI for details.†

Next, we mixed a background DNA (5′-NH_2_, 28-nt; **BD-3**) with a ligand DNA (5′-desthiobiotin, 28-nt; **LD-3**) at a 4 : 1 ratio. **BD-3** and **LD-3** have orthogonal primer binding sites (PBS-1 and PBS-2; Fig. 6a). Both **BD-3** and **LD-3** have a 7-base region complementary to a short anchor DNA (3′-desthiobiotin, 7-nt; **AD-3**). These DNA strands were mixed at a 4 : 1 : 1.5 ratio to form the dynamic library. After adding SA, the mixture was irradiated and the crosslinked duplexes were gel-purified for qPCR (quantitative PCR) analysis. The qPCR threshold cycle values (*C*_T_'s) were determined to calculate the initial copy numbers of **LD-3**/**AD-3** and **BD-3**/**AD-3** duplexes with their respective primers.^89,90^ In order to offset possible biases from experimental factors, the library was also subjected to the same procedure (irradiation, gel purification, and qPCR) with the control protein BSA. Fold enrichments were then calculated by comparing the results from these two selections (see the ESI for the calculation method; Fig. S2–S4†). As a result, a 12.0-fold enrichment of the high affinity **LD-3**/**AD-3** duplex was achieved (Fig. 6b), which is comparable to typical DCL-based selections.^16,18,19,91,92^ Gel analysis also directly confirmed the enrichment of the crosslinked **LD-3**/**AD-3** duplex (Fig. S5†). Moreover, in order to test the generality of our method, we conjugated another pair of ligands, theophylline and CBS to **LD-3** and **AD-3** DNA strands, respectively. Theophylline and CBS were found to synergistically bind the target of carbonic anhydrase-II (CA-II) in an ESAC library selection.^66^ After mixing with the background DNA **BD-3**, the formed dynamic library was subjected to the selection against the target CA-II and the negative control BSA with the same procedure. The results show that a 10.2-fold enrichment of the **LD-3**/**AD-3** duplex was achieved (Fig. 6c). Collectively, these results have demonstrated that the PS-based crosslinking mechanism is suitable for locking and analysing the equilibrium shift in DEDL selections.

**Fig. 7:**
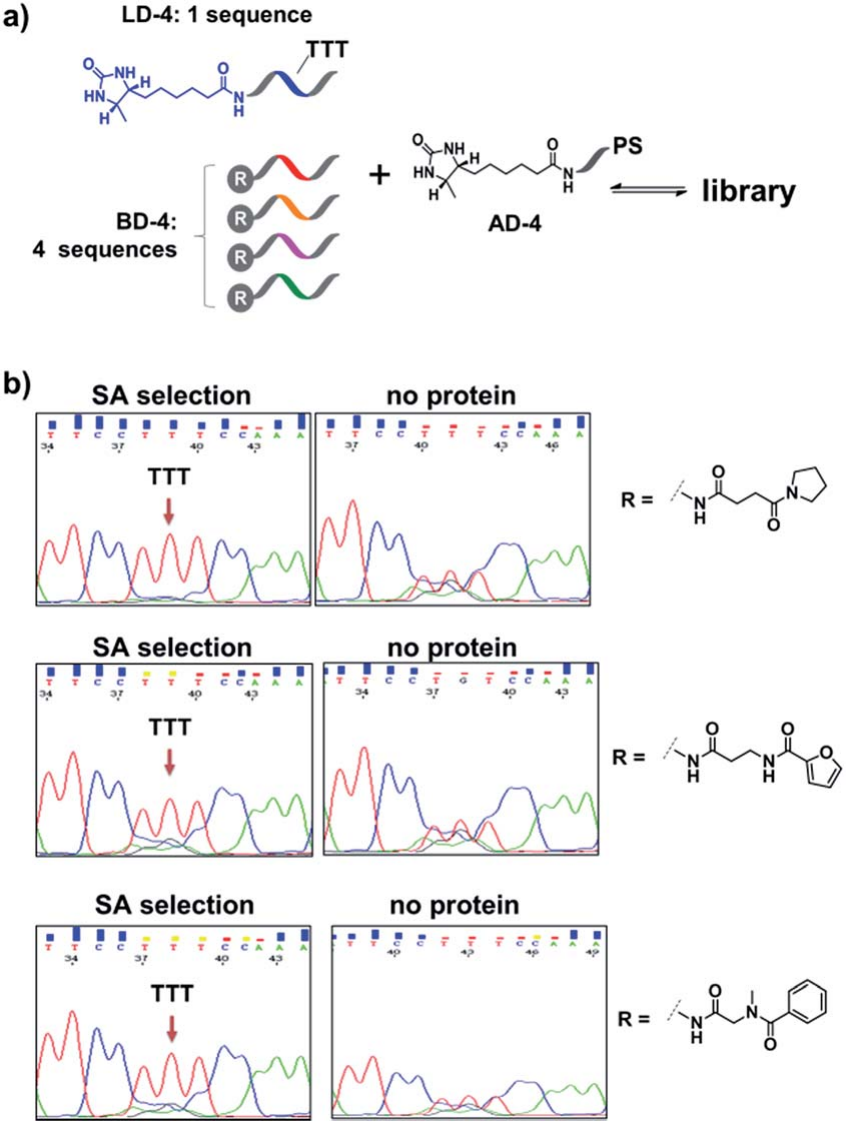
(a) Components of the model libraries. Libraries were selected against SA with the same procedure as that for Fig. 6, except Sanger sequencing was used. (b) Sequencing results; left panels: after SA selection; right panels: control selection without SA. **LD-4**: 200 nM; **BD-4**: 800 nM (total); **AD-4**: 300 nM; SA: 400 nM. See the ESI for details.†

Encouraged by these results, we further prepared several model DCLs (Fig. 7a). These libraries contain a desthiobiotin-labelled ligand DNA (**LD-4**) and 4 background (**BD-4**) DNA strands, all dynamically competing for an anchor DNA (with desthiobiotin, **AD-4**). The **BD-4** strands were also conjugated with several small molecules that are not known to bind SA, but represent typical fragment structures in a library. The ligand of desthiobiotin in **LD-4** is encoded by a “TTT” codon, while the **BD-4** strands contain varied sequences at the encoding site (“AAG”, “GCA”, “ACA” and “CGC”). These DNA strands were mixed at an equal ratio to form the library and then selected against SA. After irradiation, “hit compounds” were isolated and decoded with the same procedure as that for Fig. 6, except Sanger sequencing was used. As shown in Fig. 7b, in all cases, the “TTT” codon encoding the desthiobiotin in **LD-4** has been enriched markedly by SA due to the high affinity of the **LD-4**/**AD-4** duplex (left panels), whereas negative selections (no protein) only generated scrambled sequences at the encoding site (Fig. 7b, right panels).

**Fig. 8:**
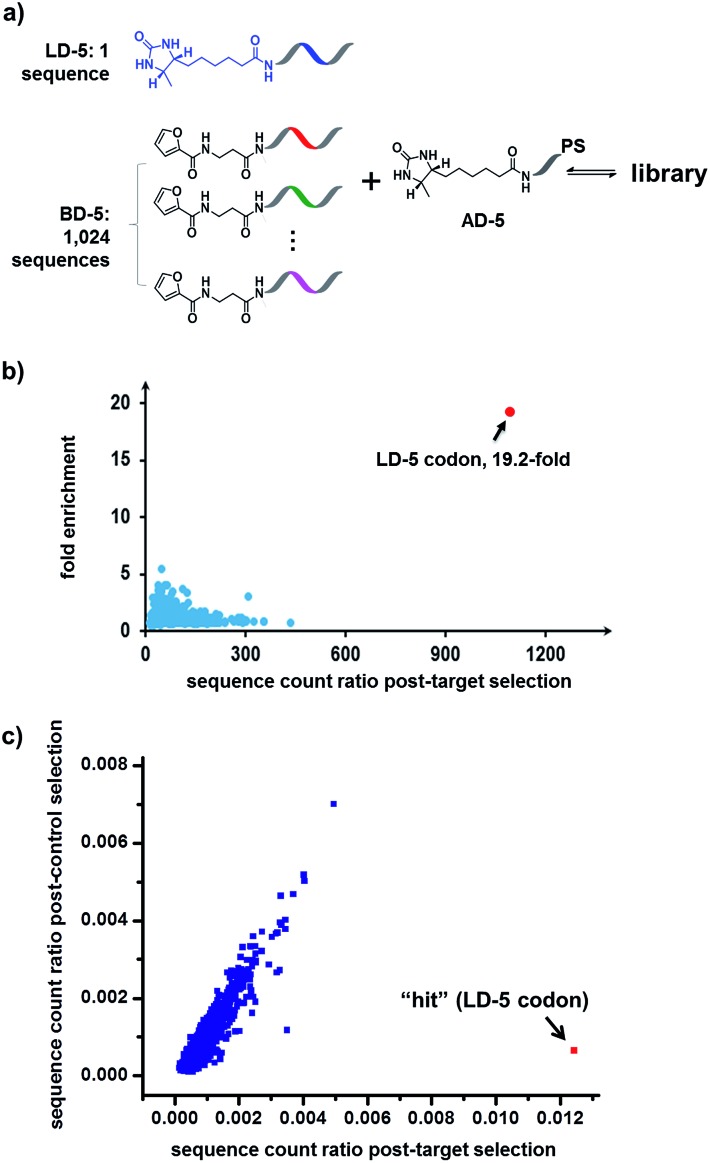
(a) Components of the 1025-member library. The library was selected against SA with the same procedure as that for Fig. 6, except Illumina® sequencing was used. (b) Plot of the fold enrichment *versus* sequence count after the target selection. Fold enrichment = (post-target selection fraction)/(post-control selection fraction). (c) Plot of sequence count ratios after the control selection (no protein added) *versus* count ratios after the target selection (with SA). Sequence ratio = (sequence count)/(total sequence count of the library). Each dot represents the DNA sequence corresponding to a library member. The “hit” containing the desired **LD-5** codon is highlighted in red. **LD-5**: 0.19 nM; **BD-5**: 200 nM (total); **AD-5**: 300 nM; SA: 400 nM. The fold enrichments for the low-count library members vary widely due to statistical under-sampling. See the ESI† for more details on the experimental procedure, data analysis and further discussion of the sequencing results.

Finally, in order to mimic library diversity, we prepared a model DCL containing 1024 background (**BD-5**) DNA strands, a ligand DNA (with desthiobiotin, **LD-5**), and an anchor DNA (with desthiobiotin, **AD-5**) (Fig. 8). The **LD-5** and all **BD-5** strands were mixed at an equal ratio, realizing a 1024-fold excess of background DNA strands relative to **LD-5**. This library was selected against the target SA and also subjected to a “no-protein” control selection, similar to that for Fig. 6, to control for biases from the selection procedures (irradiation, gel purification, PCR, sequencing, *etc.*). The selection results were decoded by high throughput DNA sequencing (Illumina®). The fold enrichments of selected sequences were plotted against the sequence counts to identify “hit compounds” (Fig. 8b). Again, due to the high affinity of the **LD-5**/**AD-5** duplex, the sequence that encodes **LD-5** was distinctly enriched (19.2-fold). In addition, the expected “hit”, **LD-5**, shows a high sequence count ratio after the target selection, while having an average count ratio in the control selection, further confirming its target specificity (Fig. 8c and S6†). It is worth noting that the wide distribution of sequence counts in both the target and control selections indicates that sufficient sequencing depth and high library synthesis quality (even distribution of library members)^93^ are both important in library selections. Although this model library only has a limited chemical diversity, these results have demonstrated our approach's suitability for the selection of large dynamic libraries.

## Conclusions

In conclusion, we have developed a DNA-encoded dynamic library (DEDL) approach for the preparation and selection of large dynamic libraries. Notably, we introduced a novel locking mechanism, which is able to take a “snapshot photo” of the library equilibrium altered by the target protein, thereby enabling the downstream hit isolation and identification. Second, our method eliminated the requirement of target immobilization and physical washing; therefore, target-induced perturbation of the library equilibrium is better preserved, and unmodified, non-immobilized proteins can be used as targets.^69,70,90^

However, the present method only encodes one fragment and thus is limited to the “affinity maturation” of known ligands (the “anchor”),^66,71,72^ rendering it unsuitable for the *de novo* discovery of synergistic fragment combinations.^38^ In contrast, nucleic acids have previously been successfully used as templates to pair DNA/PNA-linked small molecule ligands, therefore enabling the selection of synergistic fragment pairs for biological targets,^50,52,53,57–64^ and the strategy of interstrand code-transfer also realized the dual-pharmacophore ESAC libraries.^65^ These elegant studies highlight the importance of further development of dual-display DNA-encoded dynamic library,^8,94^ which indeed is currently being pursued in our laboratory using an alternative DNA architecture, more efficient crosslinker,^95^ and different decoding scheme.^96^ We will report the results in due course.
