# Supplementary material for: Design, preparation, and selection of DNA-encoded dynamic libraries
Source: Chem Sci. 2015 Sep 11;6(12):7097–104. doi: 10.1039/c5sc02467f (PMC5510007; doi:10.1039/c5sc02467f)
Supplement: Supplementary file 1 [file SC-006-C5SC02467F-s001.pdf]

Electronic Supplementary Material (ESI) for Chemical Science.
This journal is © The Royal Society of Chemistry 2015

Supporting Information

Design, Preparation, and Selection of DNA-encoded Dynamic Libraries

Gang Li,[a,‡] Wenlu Zheng,[b,‡] Zitian Chen,[c,‡] Yu Zhou,[b] Yu Liu,[a] Junrui Yang,[c] Yanyi Huang,[c]

Xiaoyu Li*[,a,b]

[a] *Key Laboratory of Bioorganic Chemistry and Molecular Engineering of the Ministry of Education, Beijing National Laboratory of Molecular Sciences (BNLMS), College of Chemistry and Molecular Engineering, Peking University, 202 Chengfu Road, Beijing, China 100871*
[b] *Key Laboratory of Chemical Genomics, School of Chemical Biology and Biotechnology, Peking University Shenzhen Graduate School, Shenzhen, China 518055*
[c] *Biodynamic Optical Imaging Centre (BIOPIC) and College of Engineering, Peking University, Beijing, China 100871*
‡ *These authors contributed equally to this work.*

E-mail: xiaoyuli@pku.edu.cn

Table of Contents

1. Abbreviations. S3

2. Materials and general methods. S5

3. Experiment conditions for the assembly and analysis of DNA-encoded dynamic library. S6

4. Modified DNA structures, sequences, and characterization. S7

5. DNA synthesis. S11

6. MS characterization of the crosslinking products (Figure 5c): S15

7. qPCR and enrichment fold calculation: S16

8. Direct gel validation of the equilibrium shift by PS-based photo-crosslinking:……………………S19

9. Analysis and discussion of the Illumina sequencing data in Figure 8:……………………..……S20

1. Abbreviations.

AMA: 1:1 (v:v) aqueous methylamine (40% wt.) : aqueous ammonium hydroxide (30% wt.)

BSA: bovine serum albumin

CA-II: carbonic anhydrase-II

CBS: 4-sulfamoylbenzoic acid

CPG: controlled-pore glass

DABCYL: (E)-4-((4-(dimethylamino)phenyl)diazenyl)benzoic acid

DB: desthiobiotin

DCC: *N*, *N*'-dicyclohexyl carbodiimide

DCM: dichloromethane

DIPEA: *N*, *N*'-diisopropylethylamine

DMF: *N*, *N*'-dimethyl formamide

DMSO: dimethyl sulfoxide

DMT: di-(4-methoxyphenyl)phenylmethyl

EDC: 1-ethyl-3-(3-dimethylaminopropyl) carbodiimide hydrochloride

FAM: carboxyfluorescein

Fmoc: 9-fluorenylmethyloxycarbonyl

HBTU: *O*-benzotriazol-1-yl-*N*, *N*, *N*', *N*'-tetramethyluroniumhexafluorophosphate

HOBt: *N*-hydroxylbenzotriazole

3-HPA: 3-hydroxybenzotriazole

MMT: 4-methoxytrityl

NHS: *N*-hydroxysuccinimide

PAGE: polyacrylamide gel electrophoresis

PBS: phosphate buffered saline

PC: photo-crosslinker

PS: psoralen

SA: streptavidin

SE: succinimidyl ester

SM: small molecule

TBE: tris-borate-EDTA

TCA: trichloroacetic acid

TEA: triethylamine

TEAA: triethylammonium acetate

2. Materials and general methods.

Unless otherwise noted, all reagents and solvents were purchased from commercial sources and used as received. Streptavidin and BSA were purchased from Sangon Biotech; CA-II was purchased from Sigma-Aldrich; Estrogen receptor (ER) alpha was purchased from ThermoFisher Scientific. Biotin, desthiobiotin and iminobiotin were purchased from Sigma-Aldrich. Theophylline-7-acetic acid was purchased from Alfa Aesar. 4-sulfamoylbenzoic acid (CBS) was purchased from J&K Scientific Ltd. Fluorescent dyes were purchased from Tianjin Heowns Biochemical Technology Co. Ltd. (DABCYL) and Beijing Fanbo Biochemicals (5, 6-FAM and 6-FAM-SE). Other common chemicals and reagents were purchased from Beijing Ouhe Technology Co. Ltd. and J&K Scientific Ltd. qPCR Supermix was purchased from Beijing Transgen Biotech. Other PCR reagents and agarose gel loading markers were purchased from Beijing Liuhe Tong Trade Co. Ltd. Water was purified with a Thermo Scientific Barnstead Nanopure system. Oligonucleotides were synthesized on standard CPG (1000 Å) beads by an automated Applied Biosystems 394 synthesizer following instrument's built-in programs. Standard phosphoramidites, other synthesis reagents and solvents were purchased from Hai Phoenix Technology and Glen Research. Anhydrous acetonitrile was freshly distilled over P_2O_5 prior to use. DNA oligonucleotides were purified by reverse-phase HPLC (Agilent 1200) using a gradient of acetonitrile (5-80%) in 100 mM TEAA (pH 7.0). DNA oligonucleotides were analyzed and purified by denaturing TBE-Urea PAGE gel or native TBE PAGE gel (18% or 20%). Samples for Illumina sequencing were analyzed by agarose gel electrophoresis, stained with ethidium bromide. All gel images were captured by a Bio-Rad Chemidoc system. Photo-crosslinking experiments were conducted by a UVP CL-1000L Ultraviolet crosslinker at 365 nm wavelength with an intensity of approximately 100 μJ /cm^2 or Uvata UV LED point light system. Concentrations of oligonucleotides were determined by the absorbance and extinction coefficient at 260 nm. Concentrations of proteins were determined by the absorbance and extinction coefficient at 280 nm.

3. Experiment conditions for the assembly and analysis of DNA-encoded dynamic library.

a) FRET experiments:

- In Figure 2 and Figure 3, fluorescent DNA (5’-FAM-DNA-ligand-3’, 200 nM) or the control DNA (5’-FAM-DNA-NH_2-3’, 200 nM), and quenching DNA (5’-ligand-DNA-DABCYL-3’, 200 nM) were mixed with respective protein (SA, ER or BSA; 400 nM) in a buffer of 0.1M TAPS/0.1 M NaCl (pH = 9.0). The total solution volume was 200 µL. Samples were then incubated at 30 ºC for 1 h before fluorescence measurement with temperature control. Excitation wavelength: 494 nm; detection wavelength: 522 nm.
- In Figure 4, **LD-1** (200 nM), **AD-**1 (200 nM), and **BD-1** (1.6 µM) were mixed with SA (400 nM), BSA (400 nM), or water (no protein control) in a buffer of 0.1M TAPS/0.1M NaCl (pH = 9.0) to reach a total volume of 200 µL. Samples were incubated at 30 ºC for 1 h before fluorescence measurement with temperature control. Excitation wavelength: 494 nm; detection wavelength: 522 nm.

b) Photo-crosslinking experiments:

- In Figure 5, 6 and 7: DNA strands were mixed in a buffer of 0.1M TAPS/0.1M NaCl (pH = 9.0) at the concentrations as specified in the figure. Samples were heated at 50 ºC briefly before slowing cooling to room temperature before protein addition. The total volume of incubation mixture was 200 µL. Samples were incubated at 30 ºC for 1 h before light irradiation at 365 nm for 10 min by a UVP CL-1000L Ultraviolet crosslinker or 30s by a Uvata UV LED point light source. 20 µL 1% SDS was added and protein was denatured at 90 ºC for 10 min over a dry bath. A 66-nt fluorescent DNA was added to all solutions as the internal fluorescence control. After ethanol precipitation, recovered samples were resolved by 18% TBE-Urea denature PAGE gel (Figure 5) or 20% TBE native PAGE gel (Figure 6, 7). Product bands were excised, extracted by TE buffer, and purified by ethanol precipitation. Purified samples were directly subjected to MS characterization, PCR and/or sequencing.
- In Figure 8: experiment conditions and procedures are the same as described above except that samples were incubated at 16 ºC and 22% TBE native PAGE gel was used. The isolated crosslinking products were subjected to PCR with long Illumina primers; PCR products were resolved by 3% agarose gel and recovered by Axygen plasmid recovery kit. The products

were quantified by Nanodrop and then submitted to BIOPIC's high-throughput sequencing platform at Peking University for Illumina sequencing.

4. Modified DNA structures, sequences, and characterization.

a) General.

DNA oligonucleotides were synthesized on an Applied Biosystems 394 DNA synthesizer using standard phosphoramidite protocols and purified by C18 reverse-phase HPLC with aqueous 0.1 M TEAA/CH_3CN gradient on Agilent 1200 HPLC systems (Eclipse-XDB C18, 5 μM, 9.4 x 200 mm or 4.6 x 150 mm). After solid-phase synthesis, oligonucleotides on CPG were cleaved by AMA (55 ºC, 55 min) over a dry bath, then concentrated, neutralized (2.0 M TEAA) before HPLC purification. For all non-standard phosphoramidites (other than standard dA-, dT-, dC- and dG-phosphoramidites), coupling time was modified to 999 seconds. Non-standard phosphoramidites were purchased from Glen Research and Wuhu Huaren. Oligonucleotides were quantitated by a BioTek Epoch UV-Vis spectrometer based on extinction coefficient at 260 nm. Oligonucleotides were characterized by either a Bruker APEX IV (for ESI-MS) or a Bruker ultrafleXtreme [for MALDI-MS, matrix: 9:1 (50 mg/mL 3-HPA in 1:1 water: acetonitrile): (50 mg/mL ammonium citrate in water)] mass spectrometer. All DNA sequences are written in 5'- to 3'- orientation unless otherwise noted.

b) Chemical structures of DNAs.

We used the standard 3'-amino-modifier C7 CPG and the 5'-amino-modifier 5 in DNA synthesis to introduce amino functional groups at the ends of DNA and various modifications (FAM, DABCYL, small molecule ligand, etc.) were coupled to DNA via these amine groups, either in solution or directly on solid phase. The PS group was introduced by using the commercially available psoralen-C2-phosphoramidite. Detailed preparations are described in Section 5. Representative chemical structures of modified DNAs are shown below:

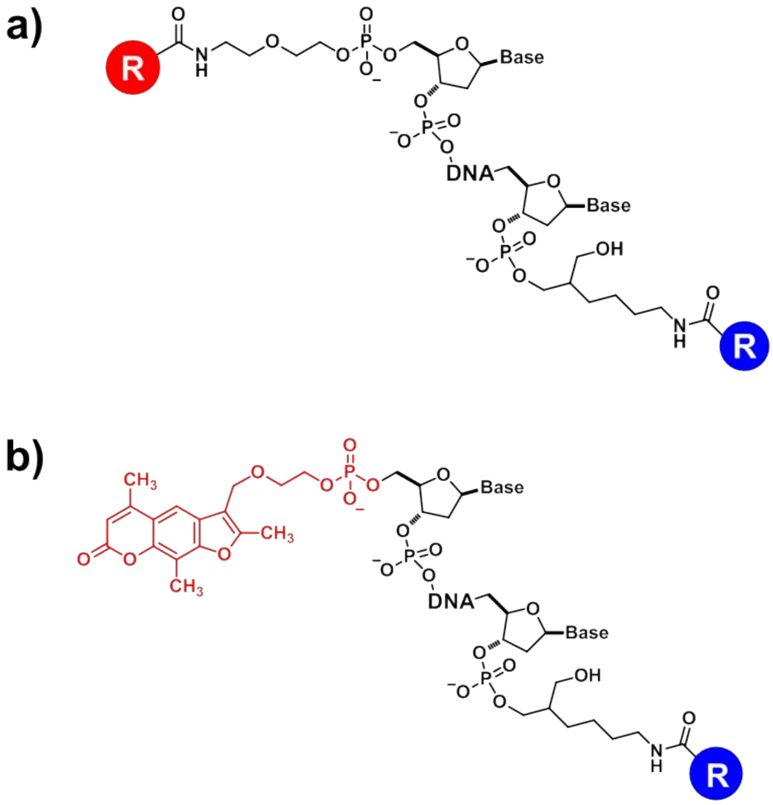

Figure S1: Representative structures of modified DNAs: a) modification via 5'- and 3'- amino modifiers; b) 5'-psoralen-modified DNAs.

c) DNA sequences.

- **Sequences in Figure 2, 3, 4:**

  5'-FAM-GTC TGC-3'-ligand (**FD**, **LD-1**)

  5'-ligand-GCA GAC T-3'-DABCYL (**QD**, **AD-1**)

  5'-GTC TGC-NH_2-3' **(BD-1)**

- **Sequences in Figure 5:**

  AD-2: 5'-PS-TAT CAA C-ligand-3'

  Fully matched **LD-2** (Figure 5b): 5'-GTT GAT ATT TTC CCG AAA GTA GAT AGG CGA-3'

Partially mismatched **LD-2** (Figure 5b): 5'-GTA GAC ATT TTC CCG AAA GTA GAT AGG CGA-3'

Fully mismatched **LD-2** (Figure 5b): 5'-CAA ATG TTT TTC CCG AAA GTA GAT AGG CGA-3'

LD-2 (Figure 5c): 5'-ligand-GTT GAT ATC CGC AGA TAG GCG ACA-FAM-3'

66-nt DNA loading control: 5'-CTT TCA GAC ATT CTT GAA CCT TCT CAC ATC TTG CTA TTC ACT TAC TGC TTA TCG TTA CTC ATT GTT-3'

- **Sequences in Figure 6:**

  AD-3: 5'-PS-TAT CAA C-ligand-3'

  LD-3: 5'-ligand-GTT GAT ATT CCC GAA AGT AGA TAG GCG A-3' (for desthiobiotin)

  LD-3: 5'-ligand-GTT GAT ATT CCC GAA AGT AGA TAG GCG T-3' (for CBS)

  BD-3: 5'-NH_2-GTT GAT ATT CCC GAA AGT ACA TTG GGC A-3'

  Forward Primer: 5'-TAG AGT CCC GAA AGT-3'

  Reverse Primer for **LD-3**: 5'-TTC TGT CGC CTA TCT-3'

  Reverse Primer for **BD-3**: 5'-TAG TCT GCC CAA TGT-3'

- **Sequences in figure 7:**

  AD-4: 5'-PS-TAT CAA C-ligand-3'

  LD-4: 5'-GTT GAT ATC CTG AAT TCC <u>TTT</u> CCA AAC TGC C-3'

  BD-4-1: 5'-GTT GAT ATC CTG AAT TCC <u>AAG</u> CCA AAC TGC C-3'

  BD-4-2: 5'-GTT GAT ATC CTG AAT TCC <u>GCA</u> CCA AAC TGC C-3'

  BD-4-3: 5'-GTT GAT ATC CTG AAT TCC <u>ACA</u> CCA AAC TGC C-3'

  BD-4-4: 5'-GTT GAT ATC CTG AAT TCC <u>CGC</u> CCA AAC TGC C-3'

  Forward Primer: 5'-AAT GAT ACG GCG ACC ACC GAG ATC TAC ACT CTT TCC CTA CAC GAC GCT CTT CCG ATC TCC TGA ATT CC-3'

  Reverse Primer:5'-CAA GCA GAA GAC GGC ATA CGA GAT GCT GTA GTG ACT GGA GTT CAG ACG TGT GCT CTT CCG ATC TGG CAG TTT GG-3'

- **Sequences in Figure 8:**

AD-5: 5'-PS-TAT CAA C-DB-3'

LD-5: 5'-ligand-GTT GAT ATC CTG AAT TCC TGA CGG GTC CAA ACT GCC-3'

BD-5: 5'-GTT GAT ATC CTG AAT TCC NCN ANC NNC CAA ACT GCC-3'

(N = A, C, G, C)

Forward Primer: 5'-AAT GAT ACG GCG ACC ACC GAG ATC TAC ACT CTT TCC CTA CAC GAC GCT CTT CCG ATC TCC TGA ATT CC-3'

Reverse Primer for **LD-5**: 5'-CAA GCA GAA GAC GGC ATA CGA GAT TCG AAG GTG ACT GGA GTT CAG ACG TGT GCT CTT CCG ATC TGG CAG TTT GG-3'

Reverse Primer for **BD-5**: 5'-CAA GCA GAA GAC GGC ATA CGA GAT TCG GGA GTG ACT GGA GTT CAG ACG TGT GCT CTT CCG ATC TGG CAG TTT GG-3'

d) DNA characterization.

oligonucleotide	expected mass (Da)	observed mass (Da)
FAM-DNA-biotin in Figure 2, **LD-1**	2742.53	2742.12
FAM-DNA-desthiobiotin in Figure 2, **FD**, **LD-1**	2712.57	2712.23
FAM-DNA-iminobiotin in Figure 2, **LD-1**	2741.55	2741.18
FAM-DNA-raloxifene in Figure 2, **LD-1**	3017.57	3019.11
DABCYL-DNA-biotin in Figure 2, **AD-1**	2957.67	2957.39
DABCYL-DNA-desthiobiotin in Figure 2, **QD**, **AD-1**	2927.71	2927.23
DABCYL-DNA-iminobiotin in Figure 2, **AD-1**	2956.68	2956.16
DABCYL-DNA-raloxifene in Figure 2, **AD-1**	3232.72	3234.56
FAM-DNA-NH_2 in Figure 2	2731.60	2731.94
**AD-2**, **AD-3**, **AD-4**, **AD-5** (desthiobiotin)	2832.65	2833.17
**AD-3 (CBS)**	2819.53	2860.62
**LD-2** in Figure 5c	8328.52	8329.00
**LD-2**, matched	9285.06	9286.50
**LD-2**, partially mismatched	9279.07	9280.10

**LD-2**, fully mismatched	9254.05	9255.20
**LD-3**, theophylline	9050.55	9058.90
**LD-5**	11368.98	11369.43

5. DNA synthesis.

a) 5'-FAM-DNA-3'-SM.

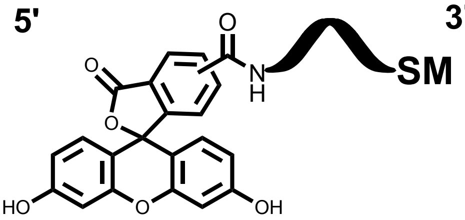

After solid phase DNA synthesis with the 3'-amino-modifier C7 CPG and 5'-amino-modifier 5, the 5'-MMT group was removed to expose the 5'-amine with 3% TCA. In a separate vial, 5, 6-FAM (15.0 mg, 40 μmol) was dissolved in 200 μL anhydrous DMF with 1.0 equiv. HBTU (15.2 mg, 40 μmol), 1.0 equiv. HOBT (5.4 mg, 40 μmol) and 2.3 equiv. DIPEA (15.2 μL, 92 μmol). After 1 hour incubation at room temperature, the activation mixture was added to the CPG. The suspension was incubated at 37 ºC overnight with agitation. The CPG was washed with DMF (3x 600 μL), CH_3CN (3x 600 μL), H_2O (3x 600 μL), CH_3CN (3x 600 μL) and then dried by gentle air stream. The 5'-FAM-labeled DNA was cleaved from CPG by AMA, desalted by a NAP-10 column (GE Pharmacia), and then purified by HPLC.

Biotin-SE (4.1 mg, 12 μmol) or desthiobiotin-SE (3.7 mg, 12μmol) was dissolved in 40 μL DMSO and then added to the 5'-FAM-labeled DNA (in 80 μL phosphate buffer, 0.25 M, pH 7.2) with sonication at 37 ºC for 2 hours. The mixture was desalted by a NAP-5 column (GE Pharmacia) and the product was purified by HPLC. For the iminobiotin, *in situ* activation was employed. Iminobiotin (12.2 mg, 50 μmol), EDC (9.6 mg, 50 μmol) and NHS (5.8 mg, 50 μmol) were dissolved in 150 μL DMSO. The mixture was incubated at 4 ºC overnight with agitation. The activated product was then added to the 5'-FAM-labeled DNA solution with sonication at 37 ºC

for 1 hour. After brief centrifugation, the mixture was separated by a NAP-5 column (GE Pharmacia) and the product was purified by HPLC.

Raloxifene was synthesized according to a published protocol (*Chem. Res. Toxicol.* **2005**, *18*, 1485-1496). The “uncleavable linker”, bis(2,5-dioxopyrrolidin-1-yl)glutarate (0.7 mg, 2.1 μmol), and raloxifene (2.3 mg, 5.6 μmol) were dissolved in 20 μL DMSO, respectively. The 5,6-FAM-labeled DNA was dissolved in 40 μL water and 40 μL phosphate buffer (~ 500 μM). The DNA solution was placed in a sonicator at 40 ºC before the uncleavable linker was added. After 2.5 minutes, the raloxifene solution was added to the mixture. The reaction was then maintained at 40 ºC for 1 hour. The mixture was desalted by NAP-5 (GE Pharmacia) and the final product was purified by HPLC.

b) 5’-SM -DNA-3’- DABCYL.

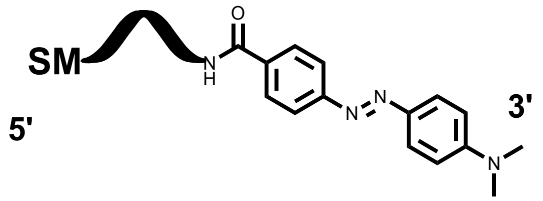

The Fmoc protecting group on the 3’-amino-modifier C7 CPG was removed by 20% piperidine in DMF. DABCYL (10.8 mg, 40 μmol) was dissolved in 400 μL anhydrous DMSO with 1.0 equiv. HBTU (15.2 mg, 40 μmol), 1.0 equiv. HOBT (5.4 mg, 40 μmol) and 2.3 equiv. DIPEA (15.2 μL, 92 μmol). After 1 hour incubation at room temperature, the activation mixture was added to the CPG. The suspension was incubated at 37 ºC overnight with agitation. The CPG was washed with DMF (3x 600 μL), CH_3CN (3x 600 μL), H_2O (3x 600 μL), CH_3CN (3x 600 μL) and then dried by gentle air stream. The DABCYL-labeled CPG was used for automated DNA synthesis. After removing the MMT protecting group by TCA on solid phase, DNA was cleaved, desalted by a NAP-10 column (GE Pharmacia), and purified by HPLC. The following labeling of biotin, desthiobiotin, iminobiotin, and raloxifeneat the 5’-end was the same as described above in a).

c) 5’-PS-DNA-3’-small molecule.

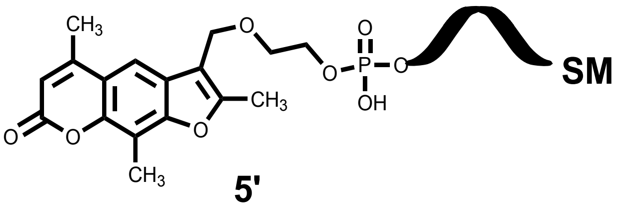

Automated DNA synthesis was performed with the 3’-amino-modified C7 CPG. Psoralen-C2-phosphoramidate (35.0 mg, 0.07 M) was dissolved in 1 mL redistilled anhydrous acetonitrile and then used as the last base. The coupling time was modified to 999 seconds and repeated twice. After cleavage, desalting, and HPLC purification, the 5’-PS-modified DNA was obtained.

Desthiobiotin was labeded at the 3’-end of the 5’-PS-modified DNA with the same method as described in a). As for the CBS modification, 5’-PS-modifed DNA was dissolved in 40 μL phosphate buffer (pH = 7.2) and CBS-NHS ester (3.6 mg, 12 μmol) was dissolved in 40 μL anhydrous DMSO. The solutions were mixed and the reaction was incubated with sonication at 37 °C for 1 hour. After brief centrifugation, the reaction mixture was desalted by a NAP-5 column (GE Pharmacia) before the product was purified by HPLC. The product was lyophilized and quantitated by a UV spectrometer and characterized by MALDI-TOF mass spectrometry.

d) LD-2 (5’- desthiobiotin-DNA-3’- FAM).

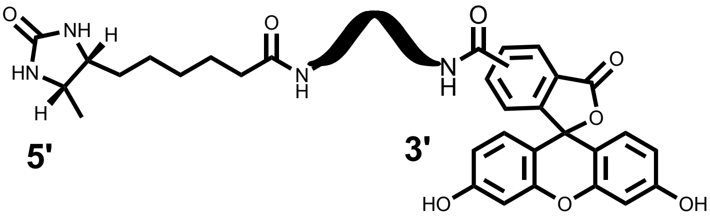

Automated DNA synthesis was performed with the 3’-amino-modified CPG. The 5’-MMT was deprotected with 3% TCA. Desthiobiotin (10.7 mg, 50 μmol) was dissolved in 200 μL anhydrous DMF with 1.0 equiv. HBTU (18.9 mg, 50 μmol), 1.0 equiv. HOBT (6.1 mg, 45 μmol) and 2.3 equiv. DIPEA (20.0 μL, 115 μmol). After 1 hour incubation at room temperature, the

activation mixture was added to CPG. The suspension was incubated at 37 ºC overnight with agitation. The CPG was washed with DMF (3x 600 μL), CH_3CN (3x 600 μL), H_2O (3x 600 μL), CH_3CN (3x 600 μL) and then dried by gentle air stream. The desthiobiotin-labeled DNA was cleaved from CPG, desalted by a NAP-10 column (GE Pharmacia), and then purified by HPLC.

FAM-SE (2.4 mg, 5.0 μmol) was dissolved in 50 μL DMF and then added to the 5'-desthiobiotin-labeled DNA (in 60 μL phosphate buffer, 0.33 M, pH 7.2) with sonication at 37 ºC for 1 hours. The mixture was desalted by a NAP-5 column (GE Pharmacia) and the product was purified by HPLC.

e) LD-3, LD-4, LD-5 (5'- desthiobiotin-DNA-3').

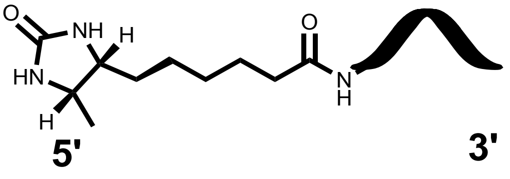

Automated DNA synthesis was performed with the 5'-amino-modifier 5. The DNA was cleaved and purified by HPLC. After MMT removal by 80% acetic acid was employed to remove the MMT protecting group, the DNA was purified again by HPLC. The labeling of desthiobiotin at the 5'-amine is the same as described above in a).

f) LD-3 (5'-theophylline-DNA-3')

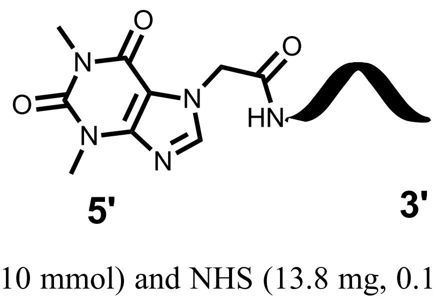

The theophylline (23.7 mg, 0.10 mmol) and NHS (13.8 mg, 0.12 mmol) were dissolved in 1.0 mL DMF. EDCI (28.7 mg, 0.15 mmol) was added to the system at room temperature. After the

mixture was stirred for 12 h, the solvent was removed by a rotary evaporator. After dissolving the residue with 10 mL chloroform, the organic phase was washed with 5 mL 2.5% $NaHSO_4$ (three times). After drying with anhydrous sodium sulfate, the crude product was obtained after the solvent was removed.

The 5'-amino modified DNA was dissolved in 12 μL sodium carbonate buffer (pH = 9.0, 0.5 M). Theophylline-NHS (0.67 mg, 2 μmol) dissolved in 100 μL DMSO was added to the system and the reaction was incubated for 12 hours. After ethanol precipitation, the pellet was purified by HPLC to obtain the product.

g) BD-4 (5'-SM-DNA-3').

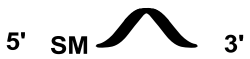

The synthesis procedure is the same as in e), except either the small molecule was activated *in situ* by EDC/NHS or the crude activated ester (SM-SE) was used in coupling with DNA.

h) 1,024 background DNA (BD-5's).

Based on the relative reactivity of different phosphoramidites (dA/dC/dG/dT = 3: 3: 2: 2.4), we mixed appropriate quantity of reagents to final concentration 0.1 M in anhydrous CH_3CN. The mixed phosphoramidite solution was used at the five "N" positions (shown above in Section 4c). The labeling of small molecule was the same as described in f).

6. MS characterization of the crosslinking products (Figure 5c):

- **DNA sequence:**

  5'-NH_2-GTT GAT ATT CCC GAA AGT ACA TTG GGC A-3'

  5'-PS-TAT CAA C-NH_2-3'

- **MS results:**

DNA	Expected MS	Observed MS
**Upper bands**	11217.97	11211.54
**Lower bands**	11217.97	11217.78

7. qPCR and enrichment fold calculation:

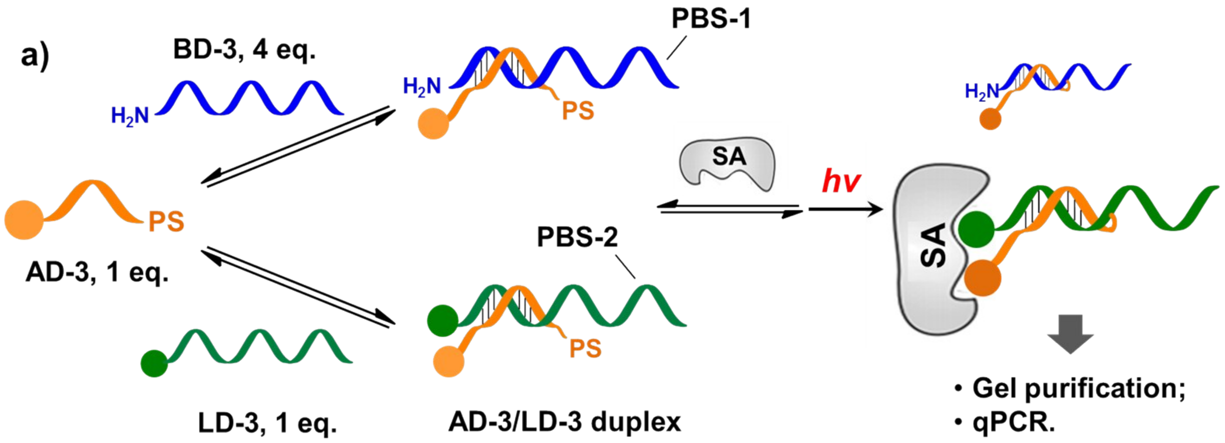

b)

	$C_{T\ (primer\ 1)}$	[AD-3/LD-3]/μM	$C_{T\ (primer\ 2)}$	[AD-3/BD-3] /μM	enrichment fold
SA	14.9	11.0	12.4	50.4	12.0
BSA	13.1	39.2	14.4	15.0	

Figure S2: a) PS-based photo-crosslinking locks the shifted equilibrium for the subsequent hit isolation and qPCR analysis. b) After gel-purification of crosslinked duplexes, qPCR was performed to determine C_T values and to calculate enrichment fold of the AD-3/LD-3 and AD-3/BD-3 duplexes.

We utilized the quantitative feature of qPCR (amplification cycle threshold value, C_T) to determine the initial copy number of amplifiable DNA templates in each experiment. We installed orthogonal PCR primers on ligand DNA and background DNA, respectively (Figure S2a).

First, for each set of primers, we conducted a series of PCR experiments with different concentrations of standard template (10 nM, 1 nM, 100 pM, 10 pM and 1 pM). The log values of the initial template concentrations were plotted against the threshold cycle and a linear function was fitted to the data (Figure S3 and S4).

All PCR experiments were performed in triplicates on a Bio-Rad CFX96 Real-Time PCR Detection System with SYBR Green as the detection dye. Thermal cycling lasts 40 times unless otherwise noted. The thermal cycling sequence was set as following: 95 ºC for 2 minutes, then iterated cycles of 95 ºC for 30 seconds, 50 ºC for 30 seconds, and 68 ºC for 15 seconds. All qPCR reactions were carried out with 2X Trans Start Top Green qPCR Super Mix in the buffer provided with a total volume of 20 μL containing Mg^{2+} (2 mM), dNTPs (200 mM), and primers (200 nM each). PCR templates were the psoralen-based photo-crosslinking products extracted from the PAGE gel. Typically, 1% of the extracted products were sufficient for the PCR amplification.

- For primer 1:

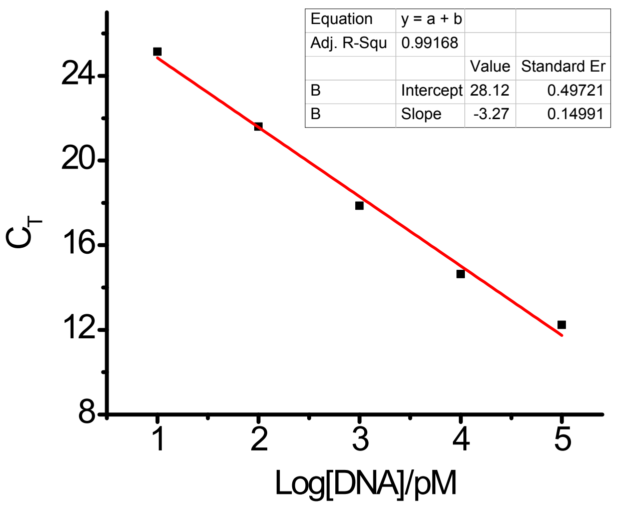

Figure S3: Standard plot of primer set #1.

From the above plot, we obtained the equation for primer set #1:

Y = -3.27 X + 28.12 (r^2 = 0.992; linear function)

- For primer 2:

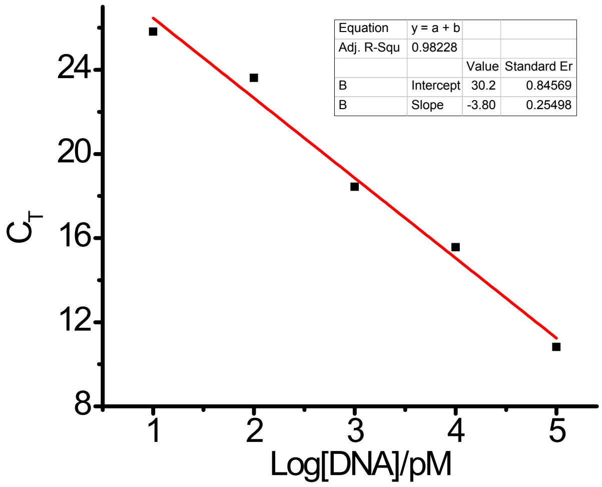

Figure S4: Standard plot of primer set #2.

From the above plot, we obtained the equation for primer set #1:

$Y = -3.80\ X + 30.27$ ($r^2 = 0.982$; linear function)

After each selection, two parallel PCR amplifications with two sets of primers were performed independently; two sets of C_T values were determined and used to calculate initial copy numbers of respective DNA duplexes. Since the process of irradiation, gel purification, and qPCR may also generate biases, a control selection against BSA was performed and C_T values were also measured.

As shown in Figure S2b, the initial copy numbers of **AD-3/LD-3** and **AD-3/BD-3** duplexes can be calculated based on C_T values and their respective standard equations. By comparing SA to BSA, an enrichment fold of 12.0 for the higher affinity **AD-3/LD-3** duplex was obtained. The enrichment fold for the theophylline/CBS-CA-II system (Figure 6c) was calculated with the same method.

8. Direct gel validation of the equilibrium shift by PS-based photo-crosslinking.

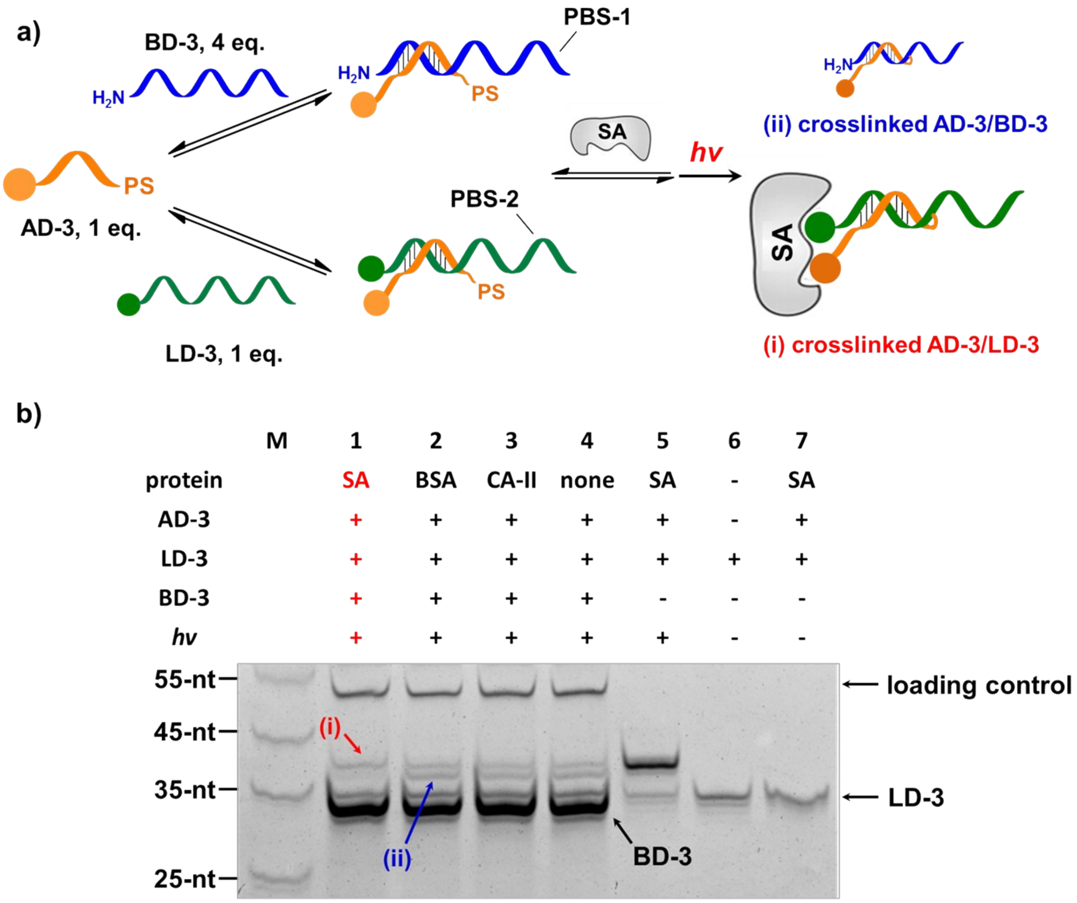

Figure S5: a) PS-based photo-crosslinking locks the shifted equilibrium and the crosslinked duplexes; (i) and (ii) were analysed by gel electrophoresis. b) Gel images of crosslinking reactions (20% TBE native PAGE). M: DNA marker; lane 1: AD-3/LD-3/BD-3 with specific target SA after irradiation; lane 2-4: same as lane 1 but with BSA, CA-II, or no protein; lane 5: standard sample of the crosslinked AD-3/LD-3 (i) duplex; lane 6: standard sample of LD-3; lane 7: AD-3/LD-3 with SA but no irradiation. LD-3: 200 nM; AD-3: 300 nM; BD-3; 800 nM; loading control: a 50-nt DNA standard. Reaction conditions and procedures are the same as Figure 6 in the main text.

Besides the qPCR analysis shown in Figure 6, we also analysed the equilibrium "freeze" and the enrichment of high affinity duplex by direct gel electrophoresis. As shown above in Figure S5, after mixing **AD-3**, **LD-3**, **BD-3** with BSA or CA-II, or with no protein added, photo-crosslinking generated two upper bands (lane 2-4). The top band is the crosslinked **AD-3/LD-3** duplex, as confirmed by the standard sample in lane 5; the bottom band is presumably the crosslinked **AD-3/BD-3** duplex. In contrast, in the presence of the specific target SA, the high affinity crosslinked

AD-3/**LD-3** duplex (**i**) was significantly enriched at the expense of the **AD-3**/**BD-3** duplex (band significantly weakened), despite the large excess of **BD-3** DNA in the mixture. This result has corroborated our qPCR analysis on enrichment shown in Figure 6 of the main text.

9. Analysis and discussion of the Illumina sequencing data in Figure 8.

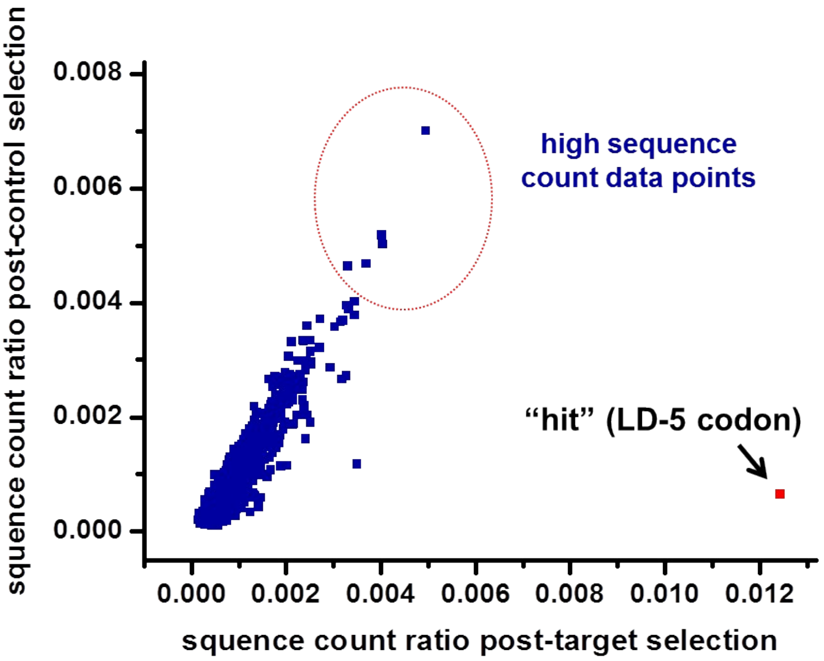

Figure S6: Plot of sequence count ratios after the control selection (no SA) versus count ratios after the target selection (with SA). The sequencing data is the same as shown in Figure 8 of the main text. “Hit” containing the desired LD-5 codon is highlighted in red. Red oval highlights several high sequence count data points. Sequence ratio = (sequence count)/(total sequence count of the library)

The control selection (no target added) can also be used to control for sequence count variations resulting from factors not related to target binding, such as non-ideal oligonucleotide synthesis (where mixed phosphoramidites were used to prepare mixed sequences and phosphoramidites may have different reactivities) and biases in PCR amplification and sequencing due to sequence difference.

In addition, a recent report by Satz has shown that library synthesis quality (even distribution of library compounds) is also important in sequencing (A. L. Satz, *ACS Chem. Biol.* 2015, **DOI:** 10.1021/acschem bio.5b00378; ref. 93 in the main text). Collectively, these observations

indicate that both sequencing depth and library quality are essential to ensure the reliability of the selection data.

The plot shown above compares the sequence count ratios of the target selection and the control selection. A linear correlation was observed, indicating those high count data points in target selection also have high counts in the control selection. It is important to always include such a data analysis in any selection to control for false results.
